# Impact of Online Racism on Suicide Ideation Through Interpersonal Factors Among Racial Minority Emerging Adults: The Role of Perceived Burdensomeness and Thwarted Belongingness

**DOI:** 10.1177/08862605221117247

**Published:** 2022-08-09

**Authors:** Brian TaeHyuk Keum

**Affiliations:** 1 University of California, Los Angeles, USA

**Keywords:** online racism, perceived burdensomeness, thwarted belongingness, suicide ideation, interpersonal theory of suicide, racial minority emerging adults

## Abstract

While a growing number of studies have documented significant links between online racism (e.g., racist interactions, contents on racial violence) and comorbid factors (e.g., depression) associated with suicide risk, no studies have examined whether online racism predicts suicide ideation and if interpersonal factors can help explain this link. Thus, the current study examined the direct relationship between online racism and suicide ideation among racial minority emerging adults, and the indirect relationships via the interpersonal factors (perceived burdensomeness and thwarted belongingness). Using data from a convenience sample of 338 racial minority emerging adults, we conducted a path analysis with online racism predicting suicide ideation through thwarted belongingness and perceived burdensomeness. Online racism significantly predicted suicide ideation via perceived burdensomeness but not thwarted belongingness. Post hoc multi-group analysis found that this pathway was consistent across Black, Asian, and Latinx groups but was completely mediated for the Asian group. The findings suggest that online racism can increase feelings of being a burden to society, which can trigger thoughts of suicide. This process may be particularly salient among Asian individuals. Implications for future research are discussed.

While suicide deaths are most frequent for those in their middle adulthood (i.e., ages 40–60) among White individuals, the highest risk of suicide deaths among racial minority groups occurs in emerging adulthood (i.e., 18–29; Arnett, 2007; Centers for Disease Control and Prevention, 2019). Specifically, from 2018 to 2019, age-adjusted suicide rates decreased for White individuals but increased for Black and Asian individuals (Ramchand et al., 2021). In fact, this increase among Black and Asian emerging adults began in 2014 (Ramchand et al., 2021). Among youth and emerging adults, American Indian and Alaskan Native individuals reported the greatest suicide rates in 2019 compared to White and other racial minority groups (Ramchand et al., 2021). These alarming trends are accompanied by studies documenting frequent suicidal behaviors among racial minority emerging adults and college students compared to White peers (Sa et al., 2020). Yet, empirical attention on racial minority youths and emerging adults’ suicide risk has been largely lacking. Addressing this health disparity requires culturally relevant investigations that center the lived experiences and adversities that contribute to suicide risks among racial minority emerging adults (Chu et al., 2010).

Against this backdrop, scholars have started to examine the role of racism as a risk factor for suicide. While the term racial minority should not assume a monolithic group that overlooks differences in racism experiences, the impact that racism can have on suicide ideation among different racial minority groups has been found to be uniform (Polanco-Roman et al., 2019). Racism denigrates and invalidates racial minority individuals in the United States based on their racial/ethnic group membership at multiple levels, including at the individual (e.g., interpersonal racial discrimination), cultural (e.g., White supremacy and cultural devaluation of people of color), and systemic levels (e.g., policies and structures that disadvantage people of color systematically). For instance, an epidemiological study with Black Americans (Oh et al., 2020) and a longitudinal study with racial/ethnic minority groups (Wang et al., 2021) have found significant associations between racial discrimination and suicide risk among racial minority youths and adults. To better understand this association, Hollingsworth et al. (2017) examined interpersonal factors that can explain the link between racial discrimination and suicide risks among Black Americans, such as perceived burdensomeness (i.e., feelings of being a burden to others in society) and thwarted belongingness (i.e., feelings of unmet social needs). However, missing in this literature is the role of online racism. Online racism has been gaining significant empirical attention as an increasingly relevant risk factor among racial minority emerging adults. Racial minority emerging adults are the most frequent users of social media and spend their time online more than their White counterparts (Auxier & Anderson, 2021). A growing body of literature on online racism has found significant links to psychosocial costs that are comorbid with suicide risk including psychological distress (Keum & Miller, 2017), depression (Tynes et al., 2008), substance use (Keum & Cano, 2021; Keum & Ahn, 2021), loneliness (Keum & Li, in press), and trauma-like symptoms (Maxie-Moreman & Tynes, 2022). Scholars indicate acute and long-term implications of these symptoms given the ongoing risk of racial minority emerging adults being exposed to traumatic content on racial violence such as widespread videos and posts of hate crimes (Maxie-Moreman & Tynes, 2022; Volpe et al., 2021). Yet, no studies have examined the link between online racism and suicide risk, and how the social and interpersonal implications of online racism may explain this link. Thus, the current study examined the direct relationship between online racism and suicide ideation among racial minority emerging adults, and the indirect relationships via the interpersonal factors (perceived burdensomeness and thwarted belongingness).

## Online Racism as a Distal Risk Factor of Suicide

Although there is knowledge about the suicide risk associated with racism, most studies to date have focused on racial discrimination conceptualized in offline settings. With the everyday salience of the internet in our lives, racism in online settings is becoming common and often portrayed in a blatant manner (Keum & Miller, 2018). By taking advantage of online factors such as online anonymity and beliefs in “digital freedom of speech,” people with racist ideologies have been taking to online platforms to share their racist views and spread hate speech without accountability (Keum & Miller, 2018). Given the polarization and amplification of online racial hate culture (e.g., White supremacist groups) and racist contents, as well as racially biased internet algorithms (e.g., search engines, image recognition), online racism has also been conceptualized to have structural implications for disproportionately harmful effects toward racial minority individuals (Volpe et al., 2021). Government and technology policies are limited in their regulation of the widespread nature of online racism on the internet, posing a serious risk as an additional dimension of social determinants that can drive health disparities among racial minority individuals. At the individual level, studies have examined the harmful costs of both interpersonal (e.g., receiving racist messages, victimization), and vicarious forms (e.g., seeing other users being victimized) of online racial discrimination (Keum & Miller, 2017; Tynes et al. 2008). Furthermore, Keum and Miller (2017) also operationalized that online racism can involve the consumption of online content that exposes systemic racism and group level practices of racial injustices in society (e.g., hate crimes, information on various systemic racial inequalities, online media dehumanizing entire cultures of racial/ethnic minority groups). Especially for today’s racial minority emerging adults who spend a considerable amount of time online daily, studies have documented the risk of being frequently and consistently exposed to racist online encounters that can have chronically stressful and socially debilitating implications over time (Tynes et al., 2008, 2020).

Coupled with the pervasive and chronically distressing nature of online racism, Carter’s (2007) race-based traumatic stress theory helps to frame online racism as a potential distal risk factor of suicide. Racial trauma theory suggests that racist encounters, particularly ones that are sudden, painful, and uncontrollable, may be perceived as a threat to the integrity and safety of the individual, resulting in adaptions and trauma-like symptoms that include hypervigilance, avoidance or numbing, and emotional distress. These responses may be survival strategies to reduce the harmful costs of racial/ethnic discrimination, but in doing so yields costs in social and psychological domains that increase susceptibility to psychopathology, including depression and suicide risk. Notably, racial discrimination (both online and offline) has been significantly associated with depressive symptoms (English et al., 2020) among Black American adolescents and substance abuse among racial minority emerging adults (e.g., alcohol; Keum & Cano, 2021) that are comorbid with the development of suicide ideation. As mental health issues such as depressive symptoms and stress are more proximal to developing suicide risk, scholars have called for more studies on the interpersonal processes of how distal risk factors like online racism could have negative social implications that can increase the risk for suicide ideation (Hollingsworth et al., 2017; O’Keefe et al., 2015).

## Online Racism and Interpersonal Risk Factors of Suicide Ideation

Durkheim’s (1963) seminal work on suicide posits that suicide could result from a lack of social bond and alienation, as well as fatalistic causes such as oppression and meaninglessness. Among more contemporary work focusing on the social perspective on suicide, the interpersonal theory of suicide (IPTS; Joiner, 2005) is particularly relevant in understanding the suicide risks from interpersonal implications of oppression such as online racism. There is more than a decade of research (Chu et al., 2017) on the IPTS with evidence suggesting that people report suicidal desires when they experience critical or negative social events that displace their fundamental need for social connection. IPTS suggests that these negative social events can cause feelings of thwarted belongingness, defined as feelings of unmet social needs, and perceived burdensomeness, defined as feelings of being a burden to others in society (Joiner, 2005). Given that these two factors characterize hopelessness, worthlessness, undesirability, and self-negativity, they are found to be proximal risk factors that can trigger people’s suicide ideation (Chu et al., 2017).

The IPTS framework has been applied to study how racial discrimination, as a form of an adverse (even trauma-like) social event, may be linked to suicide ideation through feelings of thwarted belongingness and perceived burdensomeness (Opara et al., 2020). Both feelings are likely to occur as a result of experiencing racial discrimination, as racism fundamentally denigrates racial minority individuals to feel inferior, marginalized, and ostracized from the White-dominated mainstream culture in the United States. Notably, Hollingsworth et al. (2017) found that racial microaggression experiences predicted suicide ideation among Black American young adults through perceived burdensomeness but not thwarted belongingness. This distinction is consistent with past studies identifying perceived burdensomeness to be a more robust predictor of suicide ideation in minority communities, such as sexual minority communities (Pate & Anestis, 2020), Asian American college students (Wong et al., 2011), and Latinx emerging adults (Arevalo, 2019). Hollingsworth et al. (2017) suggested that the nonsignificance of thwarted belongingness could be due to the availability of culturally relevant social support that can come to the aid of unmet social belonging, while feelings of perceived burdensomeness may be more difficult to manage given its self-deprecating and internalizing nature.

Likewise, perceived burdensomeness and thwarted belongingness may be salient factors explaining the link between online racism and suicide ideation. Given the pervasive and ubiquitous presence of online racism in social media platforms and online interactions, online racism may have pernicious and chronic social implications (Tynes et al., 2020) that can constantly reinforce the message that racial minority individuals do not belong and are a burden to society. This reinforcement is also not just at the individual level as racist views on the internet often polarize (Keum & Miller, 2018; Volpe et al., 2021) and can become a structural narrative that can overwhelm racial minority emerging adults. There is a large body of evidence suggesting that social media use is associated with lower self-esteem, worthlessness, and loneliness as people receive all kinds of denigrating messages and observe others who make them feel inferior (O’Day & Heimberg, 2021; Vogel et al., 2014). These feelings are suggestive of increased risk for suicide ideation, as Moberg and Anestis (2015) found that negative online social interactions significantly predicted thwarted belongingness. More specifically, Mitchell et al. (2018) found that cyberbullying predicted suicide ideation through depressive symptoms and thwarted belongingness. In a similar context, racist online content and interactions can function to increase feelings of thwarted belongingness and perceived burdensomeness for racial minority individuals. In fact, Keum and Li (in press) found that online racism was linked to loneliness and racism-related social avoidance among racial minority emerging adults, which may be indicative of the underlying feelings of thwarted belongingness and perceived burdensomeness. These findings, along with online racism’s links to mental health issues that are comorbid with suicide risk, suggest that thwarted belongingness and perceived burdensomeness may be important mediators to test in understanding the suicide ideation associated with online racism.

## The Present Study

**Figure 1.**
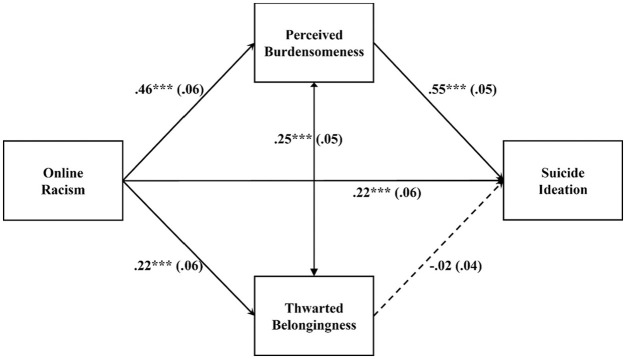
Estimated Path Model for Full Sample ***p < .001.

Emerging adulthood is a critical time when individuals may be interpersonally sensitive given their ongoing identity development and exploration of relationships with their peers (Daw et al., 2017). Especially for racial minority emerging adults without adequate social support and coping skills, adverse experiences such as online racism may increase the internalization of self-negative and self-deprecating thoughts of themselves that can give rise to suicide ideation. Thus, we examined whether online racism predicts suicide ideation and whether the two IPTS factors (perceived burdensomeness and thwarted belongingness) can explain this link among racial minority emerging adults. We employed path analysis to test the direct relationship between online racism and suicide ideation, as well as the indirect relationships through perceived burdensomeness and thwarted belongingness (parallel pathways; see Figure 1). We controlled for theoretically and statistically relevant demographic factors including COVID-19-related stress. Below were our hypotheses:

Hypothesis 1: Online racism will significantly predict suicide ideation. Greater online racism would be associated with greater suicide ideation.
Hypothesis 2: Online racism will significantly predict suicide ideation through perceived burdensomeness. Greater online racism would be associated with greater perceived burdensomeness, which would, in turn, be associated with greater suicide ideation.
Hypothesis 3: Online racism will significantly predict suicide ideation through thwarted belongingness. Greater online racism would be associated with greater thwarted belongingness, which would, in turn, be associated with greater suicide ideation.

We anticipated Hypothesis 2 to be true but our anticipation of Hypothesis 3 being true was mixed as the studies examining racism and other oppressive experiences have found perceived burdensomeness, but not thwarted belongingness, to be a robust predictor of suicide ideation (e.g., Hollingsworth et al., 2017; Wong et al., 2011). Thus, we anticipated that an alternative outcome to Hypothesis 3 could be that the indirect relationship through thwarted belongingness may not be significant. Last, we also conducted a multigroup analysis based on viable sample sizes (in our case, Black, Asian, and Latinx groups) to examine racial group differences of the hypothesized path model. Given our review suggesting that perceived burdensomeness is a robust predictor of suicide ideation among racial minority groups, we anticipated that our main findings would hold across racial groups. However, we also anticipated that there may be differences given that the context and intensity of perceived online racism in the United States may be different. For instance, Black emerging adults’ online racism experiences may be more concerned with the pervasive presence of anti-Black racist content (e.g., police brutality and hate crimes) and interactions on the internet. On the other hand, the contents of online racism may be more focused on anti-immigrant or criminalizing stereotypes of Latinx individuals while the model minority, foreigner, and COVID-19 pandemic-related stereotypes may be more salient for Asian individuals. Thus, there may be nuanced differences in how the hypothesized perceived burdensomeness and thwarted belongingness paths reflect the respective online racism experiences across the racial groups.

## Participants

A convenience sample of 338 racial minority emerging adults (*M*_age_ = 23.28, *SD* = 2.38) provided data for this study. About 57% of the participants identified as women and 43% as men. Approximately 32% of the participants identified as Black/African American, 35% as Hispanic/Latinx American, 28% as Asian/Asian American, 4% as bi/multiracial, and 1% as Native American. About 28% reported completing a bachelor’s degree, 22% some college education but no degree, 21% high school, 11% a master’s degree, 10% an associate degree, 3% a doctorate, 3% less than high school, 1% a post-baccalaureate, and 1% other. In terms of financial need, about 53% of participants reported having just enough money for their needs, 31% having not enough money to meet their needs, and 16% having more money than they need.

## Procedure

The study was approved by the Institutional Review Board (#21-000543). Participants were recruited from June to July 2021 via Qualtrics Panel Service by sending surveys to a targeted population of respondents across the nation. Participants are recruited from various sources, including website intercept recruitment, member referrals, targeted email lists, gaming sites, customer loyalty web portals, permission-based networks, social media, and so on. Participants were invited to participate in an online survey asking about their online experiences. The survey consisted of study variable measures and demographic items hosted on Qualtrics. The inclusion criteria for the study were: (1) between 18 and 29 years old, (2) self-identify as a racial minority, and (3) live in the U.S. Informed consent was provided and obtained from all participants. The survey took 15 to 20 minutes to complete and included two attention check items (e.g., “Please choose always”). All participants were compensated up to $10 in a format (e.g., cash, gift cards, rewards points, mileage points) depending on the platform they were recruited from. At the end of the survey, all participants received comprehensive mental health resources, and those who reported any suicide ideation were given specialized message with contact information for mental health professionals and a suicide hotline.

## Measures

### Online Racism

The 15-item Perceived Online Racism Scale-Short Form (PORS-SF) was used to assess people’s reports of racist online interactions and encounters with racist online content and information (Keum, 2021). The PORS-SF is a shortened version of the original 30-item PORS and retains the same three-factor structure with construct validity evidence. The three subscales are Personal Experience of Racial Cyber-Aggression subscale (five items; “I have received posts with racist comments”), Vicarious Exposure to Racial Cyber-Aggression subscale (five items; “I have seen other racial/minority users being treated like a second-class citizen”), and Online-Mediated Exposure to Racist Reality subscale (five items; “I have been informed about a viral/trending racist event happening elsewhere [e.g., in a different location]”). Participants rate each item on a 5-point Likert-type scale ranging from 1 (*never*) to 5 (*all the time*). As we are interested in assessing the collective online racism experience, we used the total scale score with higher scores indicating more frequent online racism experiences.

### Thwarted Belongingness and Perceived Burdensomeness

The Interpersonal Needs Questionnaire-15 (INQ-15; Van Orden et al., 2012) was used to assess perceived burdensomeness and thwarted belongingness. Six items assess perceived burdensomeness (e.g., “These days, I think I am a burden on society”) and nine items assess thwarted belongingness (e.g., “These days, I feel like I don’t belong”). Items are rated on a 7-point Likert-type scale ranging from 1 (not at all true for me) to 7 (very true for me) with higher scores indicating higher levels of perceived burdensomeness and thwarted belongingness. The INQ-15 has demonstrated construct validity and adequate reliability among racially diverse samples (Van Orden et al., 2012) and has been used in racial minority samples with good reliability (e.g., Arevalo, 2019; Hollingsworth et al., 2017; Wong et al., 2011).

### Suicide Ideation

We used item nine (“I have thoughts of ending my life”) of the Patient Health Questionnaire-9 (PHQ-9; Kroenke & Spitzer, 2002) to assess suicidal ideation. Participants respond on a 4-point Likert-type scale (0—*not at all* to 3—*nearly every day*) about their recent suicide ideation (past 2 weeks). Higher scores indicate a greater frequency of suicide ideation. Validity and measurement invariance of PHQ-9 with racially diverse college students has been supported (Keum et al., 2018).

### COVID-19-Related Stress

We employed a single-item question asking participants about how much stress they experienced from the COVID-19 pandemic in their day-to-day life on a 5-point Likert-type scale ranging from 0—*not at all* to 4—*very much*. The item was “How much has the COVID-19 pandemic had a stressful impact on your day-to-day life?”

### Data Analysis

A total of 830 individuals participated in the survey. Of these, 224 were removed for not meeting the inclusion criteria and thereby not providing any data; 84 were removed due to missing more than 20% of the data; and 285 were removed due to failing the attention check items. The final sample size was 338, with just one case missing 13% of the data. Little’s MCAR test was not significant, χ^2^ = 103.303, *df* = 117, *p* = .813, suggesting that data were missing completely at random. Mardia’s multivariate skewness and kurtosis test (skewness = 872.12, *z* = 75,437.97, *p* < .001; kurtosis = 5077.64, *z* = 88.20, *p* < .001) suggested nonconformity to the normality assumption (Cain et al., 2017). Thus, we used maximum likelihood estimation with robust standard errors in our analyses. We handled missing data with full-information maximum likelihood in Mplus (Enders, 2010).

We tested our hypothesized parallel mediation model (Figure 1) using path analysis in Mplus 8.7 (Muthén et al., 2017). We specified online racism as the predictor, thwarted belongingness and perceived burdensomeness as parallel mediators, and suicide ideation as the outcome. We controlled for hours online per day, COVID-19-related stress, education level, and financial need. Model fit was evaluated based on the following fit indices (Hu & Bentler, 1999): (a) the root mean square error of approximation (RMSEA; close to <0.08 for “acceptable” fit); (b) the comparative fit index (CFI) and Tucker-Lewis fit index (>0.95 for “good” fit); and (c) the standardized root mean square residual (SRMR; close to <0.08 for “acceptable” fit). To examine specific path coefficients and indirect (i.e., mediation) effects, we followed best practices (Hayes & Scharkow, 2013) and adopted the bootstrap method using 5,000 random samples. We used 95% Confidence Interval (CI) to assess the statistical significance of the mediation effects where CIs excluding 0 were deemed equivalent to *p* < .05. Based on adequate sample sizes of racial groups, we conducted a multigroup analysis to examine group differences in the hypothesized model.

## Results

Bivariate correlations, internal reliability estimates, and descriptive statistics are in Table 1. On average, participants reported spending 8.43 hours (*SD* = 5.05) online a day. The mean COVID-19-related stress was 2.17 (*SD* = 1.13). Online racism was correlated with suicide ideation and perceived burdensomeness at medium effect and with thwarted belongingness at small effect. Suicide ideation was correlated with perceived burdensomeness at medium to large effect and thwarted belongingness at small effect. About 51% indicated having no suicide ideation, 23% reported suicide ideation for several days, 15% more than half the days, and 11% nearly every day.

**Table 1.** Descriptive Statistics and Bivariate Correlations of Study Variables.

Descriptive Correlations												
Variables	Min	Max	M	SD	α	Skewness	Kurtosis	1	2	3	4	5
1. PORS	15.00	68.00	35.55	14.14	.95	0.19	−0.87					
2. SI	0	3.00	0.87	1.05	—	0.85	−0.61	.47**				
3. PB	6.00	42.00	17.78	10.91	.81	0.47	−0.97	.45**	.65**			
4. TB	9.00	63.00	33.91	11.19	.96	−0.10	0.07	.22**	.21**	.32**		
5. Hrs.On	0	24.00	8.43	5.05		1.35	2.01	.14*	.16**	.17**	.21**	
6. COVID-19 Str	0	4.00	2.17	1.13		−0.07	−0.66	.29**	.15**	.16**	.15**	.04

*Note*. PORS = Perceived Online Racism Scale; SI = suicide ideation; PB = perceived burdensomeness; TB = thwarted belongingness; Hrs.On = average hours online per day; COVID-19 Str = COVID-19-related stress.

* *p* < .05. **p < .01.

### Parallel Mediation Model With Full Sample

Figure 1 lists the standardized path estimates and Table 2 lists the path analysis results. Our hypothesized parallel mediation model fit the data well with the full sample: $\chi_{\mathrm{YB}}^{2}\;=24.202$ , *df* = 8, *p* = .002; RMSEA = 0.077 [0.025, 0.119]; CFI = 0.95; SRMR = 0.042. All control variables (hours online per day, COVID-19-related stress, education level, financial need) were not significant in predicting the dependent variable except for financial need. Overall, online racism significantly predicted suicide ideation (standardized effect β = .471, 95% bootstrapped CI = [0.369, 0.573]). The total effect was decomposed into a significant direct effect (β = .224, 95% bootstrapped CI = [0.114, 0.334]) and a significant total indirect effect through the hypothesized mediators (standardized total indirect effect β = .247, 95% bootstrapped CI = [0.171, 0.323]) that explained 52% of the total effect. The indirect pathway from online racism to suicide ideation via perceived burdensomeness was significant (standardized total indirect effect β = .252, 95% bootstrapped CI = [0.176, 0.328]). The indirect pathway from online racism to suicide ideation via thwarted belongingness was not significant (standardized total indirect effect β = −.005, 95% bootstrapped CI = [−0.023, 0.013]). These results supported our hypothesis that perceived burdensomeness, but not thwarted belongingness significantly explains the link between online racism and suicide ideation. Racial minority emerging adults who experience online racism reported feeling like a burden to society, which was in turn related to their suicide ideation. The model accounted for 47% of the variance in suicide ideation.

**Table 2.** Estimate of Indirect Effects from Bootstrap Analysis.

IV	Mediator(s)	DV	Standardized Effect Estimate	*SE*	95% Bootstrap CI
Full sample					
Total direct effect					
PORS		SI	0.224	0.06	[0.114, 0.334]
Total indirect effect					
PORS		SI	0.247	0.04	[0.171, 0.323]
Specific indirect effect					
PORS	→ PB →	SI	0.252	0.04	[0.176, 0.328]
PORS	→ TB →	SI	−0.005	0.01	[−0.023, 0.013]
Black					
Total direct effect					
PORS		SI	0.266	0.12	[0.041, 0.492]
Total indirect effect					
PORS		SI	0.188	0.07	[0.054, 0.321]
Specific indirect effect					
PORS	→ PB →	SI	0.216	0.06	[0.093, 0.338]
PORS	→ TB →	SI	−0.028	0.04	[−0.105, 0.050]
Latinx					
Total direct effect					
PORS		SI	0.305	0.10	[0.111, 0.499]
Total indirect effect					
PORS		SI	0.266	0.07	[0.124, 0.408]
Specific indirect effect					
PORS	→ PB →	SI	0.266	0.07	[0.124, 0.408]
PORS	→ TB →	SI	0.000	0.006	[−0.012, 0.012]
Asian					
Total direct effect					
PORS		SI	0.170	0.09	[−0.012, 0.352]
Total indirect effect					
PORS		SI	0.240	0.07	[0.096, 0.384]
Specific indirect effect					
PORS	→ PB →	SI	0.234	0.07	[0.090, 0.377]
PORS	→ TB →	SI	0.006	0.02	[−0.029, 0.041]

*Note*. IV = independent variable; DV = dependent variable; PORS = Perceived Online Racism Scale; SI = suicide ideation; PB = perceived burdensomeness; TB = thwarted belongingness.

### Multigroup Analysis

Our hypothesized parallel mediation model fit the data well across Black (*n* *=* 105), Latinx (*n* *=* 114), and Asian (*n* *=* 96) groups: $\chi_{\mathrm{YB}}^{2}=38.814$ , *df* = 24, *p* = .02; RMSEA = 0.077 [0.025, 0.119]; CFI = 0.95; SRMR = 0.052. All control variables were not significant in predicting the dependent variable except for financial need. Table 2 lists the path analysis results for each group.

#### Black group

**Figure 2.**
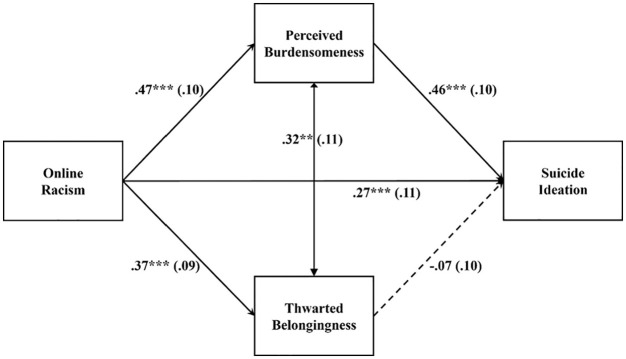
Estimated path model for the Black group. **p < .01. ***p < .001.

Figure 2 lists the standardized path estimates. Overall, online racism significantly predicted suicide ideation (standardized effect β = .454, 95% bootstrapped CI = [0.237, 0.671]). The total effect was decomposed into a significant direct effect (β = .266, 95% bootstrapped CI = [0.041, 0.492]) and a significant total indirect effect through the hypothesized mediators (standardized total indirect effect β = 0.188, 95% bootstrapped CI = [0.054, 0.321]) that explained 41% of the total effect. The indirect pathway from online racism to suicide ideation via perceived burdensomeness was significant (standardized total indirect effect β = .216, 95% bootstrapped CI = [0.093, 0.338]). The indirect pathway from online racism to suicide ideation via thwarted belongingness was not significant (standardized total indirect effect β = −.028, 95% bootstrapped CI = [−0.105, 0.050]). Perceived burdensomeness but not thwarted belongingness significantly explained the link between online racism and suicide ideation. Black emerging adults who experience online racism reported feeling like a burden to society, which was in turn related to their suicide ideation. The model accounted for 41% of the variance in suicide ideation.

#### Latinx group

**Figure 3.**
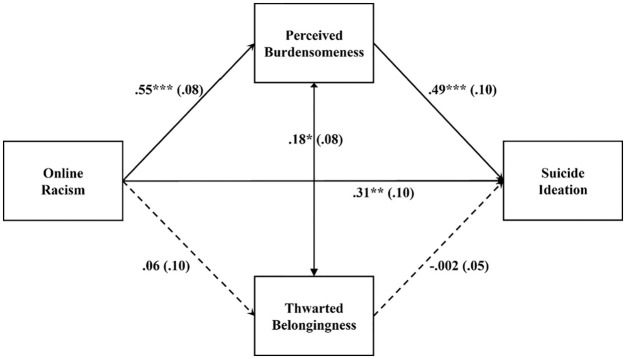
Estimated path model for the Latinx group. *p < .05. **p < .01. ***p < .001.

Figure 3 lists the standardized path estimates. Overall, online racism significantly predicted suicide ideation (standardized effect β = .571, 95% bootstrapped CI = [0.425, 0.717]). The total effect was decomposed into a significant direct effect (β = .305, 95% bootstrapped CI = [0.111, 0.499]) and a significant total indirect effect through the hypothesized mediators (standardized total indirect effect β = .266, 95% bootstrapped CI = [0.124, 0.408]) that explained 47% of the total effect. The indirect pathway from online racism to suicide ideation via perceived burdensomeness was significant (standardized total indirect effect β = .266, 95% bootstrapped CI = [0.124, 0.408]). The indirect pathway from online racism to suicide ideation via thwarted belongingness was not significant (standardized total indirect effect β = .000, 95% bootstrapped CI = [−0.012, 0.012]). Perceived burdensomeness but not thwarted belongingness significantly explained the link between online racism and suicide ideation. Latinx emerging adults who experience online racism reported feeling like a burden to society, which was in turn related to their suicide ideation. The model accounted for 51% of the variance in suicide ideation.

#### Asian group

**Figure 4.**
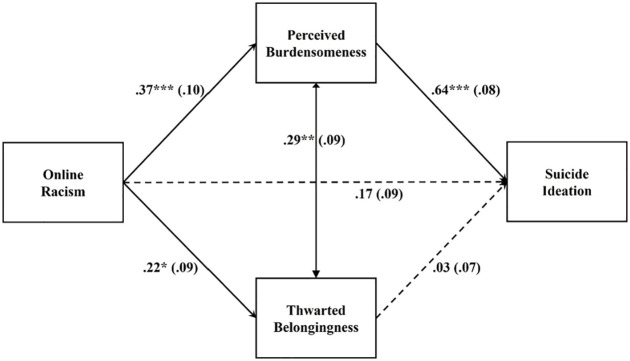
Estimated path model for the Asian group. *p < .05. **p < .01. ***p < .001.

Figure 4 lists the standardized path estimates. Overall, online racism significantly predicted suicide ideation (standardized effect β = .410, 95% bootstrapped CI = [0.222, 0.598]). The total effect was decomposed into a nonsignificant direct effect (β = .170, 95% bootstrapped CI = [−0.012, 0.352]) and a significant total indirect effect through the hypothesized mediators (standardized total indirect effect β = .240, 95% bootstrapped CI = [0.222, 0.598]) that explained 59% of the total effect. The indirect pathway from online racism to suicide ideation via perceived burdensomeness was significant (standardized total indirect effect β = .234, 95% bootstrapped CI = [0.090, 0.377]). The indirect pathway from online racism to suicide ideation via thwarted belongingness was not significant (standardized total indirect effect β = .006, 95% bootstrapped CI = [−0.029, 0.041]). Perceived burdensomeness but not thwarted belongingness significantly explained the link between online racism and suicide ideation. The nonsignificant direct effect supported the particular salience of perceived burdensomeness in explaining the suicide ideation associated with online racism. Asian emerging adults who experience online racism reported feeling like a burden to society, which was in turn related to their suicide ideation. The model accounted for 45% of the variance in suicide ideation.

## Discussion

Although growing research has examined the role of interpersonal factors in suicide risk associated with offline forms of racism (e.g., Hollingsworth et al., 2017), there is a paucity of knowledge on how online racism and interpersonal factors may elevate suicide risk among racial minority emerging adults. Given the everyday salience of online exposure to racist interactions and content in their lives (Keum & Miller, 2018), there may be significant negative interpersonal implications. The current study is the first to examine how being victimized by online racist interactions and being exposed to racist content in online settings may increase suicide ideation through feelings of thwarted belongingness and perceived burdensomeness among racial minority emerging adults. We found that online racism significantly predicted suicide ideation. Consistent with previous research on racism and suicide risk on Asian (Wong et al., 2011) and Black American individuals (Hollingsworth et al., 2017), online racism indirectly predicted suicide ideation through perceived burdensomeness but not through thwarted belongingness even though online racism significantly predicted thwarted belongingness. In line with previous speculations (e.g., Hollingsworth et al., 2017), it is possible that the feelings of thwarted belongingness may have been less relevant to triggering suicide ideation given that the vast nature of online networks and social media platforms allow individuals to connect with various groups and satisfy their unmet social needs (Oh et al., 2014). Thwarted belongingness is also based on disruptions in interrelations with others, something that may be managed better with online social connections whereas perceived burdensomeness is more of an internalizing and self-deprecating tendency that may be harder to manage even with online social support. Furthermore, this trend was consistent across Black, Latinx, and Asian groups in our sample. Interestingly, the effect size of the path estimates between perceived burdensomeness and suicide ideation was the largest for the Asian group and the direct effect was also not significant, suggesting that perceived burdensomeness seems to have the greatest salience regarding the impact that online racism can have on suicide ideation in this group.

Our findings suggest that racial minority emerging adults who experience online racism reported greater feelings of being a burden to society, which in turn was associated with greater suicide ideation. The findings raise great concern regarding the downstream severity and the potential fatality of the psychosocial impact of online racism. Given that emerging adulthood is a critical and sensitive time for identity exploration and social network development (Arnett, 2007), racial minority emerging adults may be especially made to feel like a burden to the White-dominated mainstream society when they persistently encounter racially denigrating and victimizing online interactions, contents on racial violence (e.g., hate crimes), and contents that illuminate the longstanding reality of systemic racism in the United States. The results highlight online racism as a contemporary and digitally relevant risk factor of suicide among these individuals, particularly in light of the disproportionately larger suicide deaths in this group during their emerging adulthood (Centers for Disease Control and Prevention, 2019). As Volpe et al. (2021) noted, the results also imply that online racism may be a structural inequity that can disproportionately increase the suicide risk over time among racial minority emerging adults.

It is important to note that while perceived burdensomeness was a robust predictor of suicide ideation across Black, Latinx, and Asian groups, its role seems to be the most pronounced in the Asian group. There are several potential reasons for this. First, previous studies on suicide among Asian American adults suggest that perceived burdensomeness may be particularly salient in precipitating suicide ideation in this group as it may be accompanied by shame (Wong et al., 2022). For some Asian individuals, perceiving that they have brought shame to their close friends, family, and their communities may amplify their feelings of being a burden to their loved ones and society. In fact, Wong et al. (2022) found that among suicide notes left by Asian decedents (from National Violent Death Reporting System), messages asking for forgiveness were commonly observed suggesting that absolving feelings of perceived burdensomeness may have been the main motivator for suicide. Additionally, Tang and Masicampo (2018) found that perceived burdensomeness increased Asian American college students’ suicide ideation and decreased their willingness to seek help. Thus, Asian individuals may believe that they do not deserve help given their perception of being a burden and suffer in silence while hiding their suicide ideation until they choose to act on their ideation (Chu et al., 2018).

A second reason is the context of the pandemic-related anti-Asian hate and racism. Data for this study were collected in 2021 and should be contextualized within the marked increase in pandemic-related anti-Asian sentiments across the country (Dhanani & Franz, 2020). It is likely that this sentiment was involved in much of the online racism experienced by Asian individuals in our sample. Being stereotyped and labeled as “biologically dangerous” and scapegoated for starting the pandemic (Dhanani & Franz, 2020) likely pushed Asian individuals to feel like a burden to society. In fact, we observed that the perceived burdensomeness and thwarted belongingness scores in the current sample were higher than the mean perceived burdensomeness (7.45) and thwarted belongingness (19.65) scores reported in a previous study by Hollingsworth et al. (2017). A recent study by Keum and Choi (in press) found that anti-Asian COVID racism was linked to increased depressive symptoms and alcohol use to cope with the distress among Asian American emerging adults. Given that the anti-Asian dynamic may be pervasive and explicit in the current sociopolitical climate in the United States, perceived burdensomeness may have been a robust mediator between online racism and suicide ideation among Asian individuals in our sample.

## Limitations and Future Research

Although this is the first study to examine online racism and suicide risk, it has several limitations that inform future research. First, since we used a cross-sectional design, we are not able to evaluate any causal relationships and directionality of the hypothesized relationships. Future studies with longitudinal designs should confirm our findings and extend how the development of thwarted belongingness and perceived burdensomeness can be understood over time in response to experiences of online racism. For instance, it would be important to explore how exposure to online narratives on COVID-19-related anti-Asian hate (Dhanani & Franz, 2020) could change over time for Asian Americans with long-term risk of developing mental health issues and suicide ideation. Second, while we focused on online racism, future studies could focus on both online and offline racism and examine how the interaction between the two experiences could inform the risk of suicide ideation. Third, suicidal ideation was measured using a single item. Although single item measures can be good proxy variables on suicide ideation (Jackman et al., 2021), they are limited in validity and lacks the breadth of assessment. It would be important for future studies to incorporate a more rigorous measure of suicide ideation and also include behavioral aspects (e.g., plans, attempts). Fourth, even though the single item suicide ideation measure from PHQ-9 we used demonstrated validity across racial minority college students (Keum et al., 2018), our sample was not entirely college students. Given that there may be nuanced differences in suicide ideation between college and noncollege emerging adults, future studies should examine this potential contrast. Fifth, while we focused on racial minority emerging adults, the results cannot be generalized to all racial minority groups as not all groups were present in our sample and our sample was not nationally representative. As well, even though we controlled for the average number of hours online per day, we cannot generalize our findings because social media use (differences in platforms and hours spent) may vary significantly in the population. The findings will need to be replicated with a larger, more representative sample that includes other racial minority groups such as Native Americans and Middle Eastern individuals. Future studies are also encouraged to take into consideration the varying range of social media use and differences in the sentiments of online racism (e.g., anti-Black racism, pandemic-related anti-Asian hate) based on our multigroup analysis. A larger sample would also provide more analytic power and help to confirm a more robust result on the indirect effects found in our study. Last, it would be important to explore how oppression associated with other intersecting identities (e.g., transgender, disability) could further increase the risk of suicide ideation linked to online racism. For instance, although not available in our study, future studies should explore how the online racism experiences could be compounded by online transphobia among racial minority transgender individuals. In addition to quantitative studies, qualitative studies on personal narratives and social media discourse could help illuminate these compounding experiences.

Alongside better understanding of the pathway between online racism and suicide ideation, studies are needed for intervention implications. Studies have shown that perceived burdensomeness is a modifiable predictor of suicide ideation and can be reduced via interventions (Allan et al., 2018). For example, Allan et al. (2018) found that cognitive bias modification and psychoeducation helped reduce perceived burdensomeness, which in turn reduced suicidal thoughts among a racially diverse sample. Thus, existing evidence suggests that tailored individual interventions could target online racism-related perceived burdensomeness. Additionally, at the societal and institutional level, studies need to focus on how we can encourage and promote advocacy around the issue of online racism and develop a narrative that allows racial minority emerging adults to counter and externalize any perceived burdensomeness they could develop from online racism. For example, online platforms and social media can be harnessed to counter the harmful racist content and interactions on the internet (Keum & Miller, 2018). Ultimately, continued examination of the pathway between online racism and suicide ideation alongside intervention development is paramount given that racial minority emerging adults are heavily socialized by online experiences in today’s digital society.
